# Characterizing and quantifying human movement patterns using GPS data loggers in an area approaching malaria elimination in rural southern Zambia

**DOI:** 10.1098/rsos.170046

**Published:** 2017-05-03

**Authors:** Kelly M. Searle, Jailos Lubinda, Harry Hamapumbu, Timothy M. Shields, Frank C. Curriero, David L. Smith, Philip E. Thuma, William J. Moss

**Affiliations:** 1 Department of Epidemiology, Bloomberg School of Public Health, Johns Hopkins University, Baltimore, MD, USA; 2 Macha Research Trust, Macha, Choma District, Zambia; 3 Institute for Health Metrics and Evaluation, University of Washington, Seattle, WA, USA

**Keywords:** human mobility, malaria elimination, GIS, GPS loggers, movement patterns, Zambia

## Abstract

In areas approaching malaria elimination, human mobility patterns are important in determining the proportion of malaria cases that are imported or the result of low-level, endemic transmission. A convenience sample of participants enrolled in a longitudinal cohort study in the catchment area of Macha Hospital in Choma District, Southern Province, Zambia, was selected to carry a GPS data logger for one month from October 2013 to August 2014. Density maps and activity space plots were created to evaluate seasonal movement patterns. Time spent outside the household compound during anopheline biting times, and time spent in malaria high- and low-risk areas, were calculated. There was evidence of seasonal movement patterns, with increased long-distance movement during the dry season. A median of 10.6% (interquartile range (IQR): 5.8–23.8) of time was spent away from the household, which decreased during anopheline biting times to 5.6% (IQR: 1.7–14.9). The per cent of time spent in malaria high-risk areas for participants residing in high-risk areas ranged from 83.2% to 100%, but ranged from only 0.0% to 36.7% for participants residing in low-risk areas. Interventions targeted at the household may be more effective because of restricted movement during the rainy season, with limited movement between high- and low-risk areas.

## 1. Background

Host population movement is known to contribute to the transmission of infectious diseases [1–3]. The spatial dependency and heterogeneity of infectious diseases make pathogens susceptible to spread via population movement. Transmission of vector-borne diseases is particularly heterogeneous [3–5]. For mosquito-borne diseases, specifically malaria, this heterogeneous transmission typically is owing to differential contact between humans and mosquitoes as a result of migration, overlapping activity space and unequal biting rates [4,6,7]. During the first global malaria eradication campaign in the 1950s and 1960s, failure to account for human population movement was identified as one of the factors that contributed to the programme's failure [1,3].

In areas approaching malaria elimination, human mobility patterns are important in determining the relative proportions of malaria parasites that are imported or the result of low-level, endemic transmission [8–13]. Population movement patterns can threaten malaria elimination in three primary ways [1,8]. The first is through uninfected residents travelling to higher malaria risk areas and transmitting parasites to local vectors upon returning home. The second is through infected visitors transmitting to local vectors [1,8]. The third is through infected migrants re-locating and transmitting to local vectors [8]. As human movement patterns and malaria transmission are dynamic processes, individuals can acquire malaria and then transmit while travelling or upon return to their residence [2,3]. These movement patterns occur over both long distances (i.e. between districts, provinces, states or countries) and short distances (i.e. within neighbourhoods or villages), impacting malaria transmission and potentially threatening elimination [1,12–14].

Many methods to measure human mobility have been explored to describe the impact of human movement on malaria transmission [15]. Long-distance migratory patterns have been characterized using census data on birth-place and prior residence [15,16]. Many countries include questions on recent travel in malaria indicator surveys [15]. Recent travel history also is used to classify passively detected cases as imported. In research settings, movement diaries and travel histories have been used to measure human movement [15–17]. However, these methods are subject to recall and social desirability bias, and small-scale movement is difficult to determine from survey data [15,17].

Mobile phone data are a convenient and accurate method for measuring human movement [8,15]. However, there are limitations with mobile phone data to measure human movement patterns, particularly in rural settings. The use of mobile phone data to measure movement assumes that individuals who own a mobile phone are representative of the population and that a single person uses the phone [18,19]. In many areas, multiple mobile service providers are available and individuals have different subscriber identify module cards under different provider accounts. Mobile phone coverage is limited in some rural areas [20], preventing its use in detecting movement in these settings, and cannot readily measure movement across international borders. Finally, mobile phone data are reported at a population level without individual demographic information. Mobile phone data are primarily useful for capturing long-distance movement in areas of high mobile phone coverage but do not capture short-distance human mobility patterns that impact the micro-epidemiology of malaria transmission, specifically in rural areas [8,15,18,21].

On both long- and short-distance spatial scales, human movement can be measured at a fine spatio-temporal resolution using commercially available GPS data loggers to describe individual movement patterns [22–25]. Their low cost and ease of use makes GPS data loggers ideal for tracking human movement to infer risk over specific time periods, including peak transmission seasons and vector biting times [26]. Commercially available GPS data loggers have been used to investigate individual human mobility and its impact on the transmission of several infectious diseases, including schistosomiasis, hookworm and dengue virus [22–24,26], and have been validated under different geographical and environmental conditions [27]. Commercially available GPS data loggers were used to examine movement patterns in a study of influenza at a university in New Jersey, USA [28], and of dengue in Iquitos, Peru [24,26]. However, most studies reporting data using commercially available GPS loggers were conducted in North and South America, and in urban or peri-urban settings [22,24,26,28–30]. One exception was a study conducted in northern Tanzania among the Hadza hunter–gatherer population to explore foraging patterns consistent with Lévy random walks, which involve primarily short-distance movements combined with rare long-distance movements that describe foraging searches of multiple organisms [31]. Otherwise, little is known of small-scale movement patterns in rural sub-Saharan Africa and how these patterns may impact malaria transmission, control and elimination.

Commercially available GPS data loggers were used to determine movement patterns among a population of rural, agrarian participants selected from a longitudinal cohort study of malaria epidemiology in Southern Province, Zambia. These analyses aid in explaining the micro-epidemiology of malaria transmission and the risk of imported malaria as elimination is achieved and sustained [15]. Knowledge of mobility patterns and their potential impact on malaria transmission can inform the planning of malaria elimination strategies, particularly the targeting of interventions that account for spatial and seasonal variations in mobility.

## 2. Methods

### 2.1. Study site and population

The study was conducted in the rural catchment area of Macha Hospital in Choma District, Southern Province, Zambia, 70 km from the nearest town of Choma and approximately 1200 km^2^ in area. The single rainy season lasts from November through to April, followed by a cool dry season from April until August and a hot dry season from August through to November. Malaria transmission peaks during the rainy season with the highest incidence of clinical malaria typically occurring in April [32]. The primary vector in the area is *Anopheles arabiensis* [32,33]. The hospital catchment area is populated by villagers living in small, scattered homesteads. The parasite prevalence, measured by active surveillance, declined in this area over the past decade, from 9.2% in 2008 to less than 1% in 2013 [34]. Artemisinin combination therapy with artemether–lumefantrine was introduced as first-line anti-malarial therapy in Zambia in 2002 [35,36] and into the study area in 2004. In Zambia, long-lasting insecticide-treated nets (LLINs) are distributed through antenatal care clinics and additional mass distribution campaigns [37]. LLINs were widely distributed in the study area in 2007 [38] and more than 11 000 LLINs were distributed from nine health posts in the catchment area of Macha Hospital in 2012, with additional LLINs distributed in 2014 according to the Office of the Macha Hospital Environmental Health Technician.

Satellite images were used to develop a sampling frame for the random sampling of households to recruit and enrol individuals into longitudinal and cross-sectional surveys of malaria parasitaemia starting in 2008 [38]. The identification and enumeration of households was done manually to delineate household and non-household structures (kraals, schools and larger structures) [38]. Households randomly selected from the sampling frame were recruited and enrolled in one of two cohorts: cross-sectional or longitudinal. Households enrolled in the longitudinal cohort were repeatedly surveyed every two months, whereas households enrolled in the cross-sectional cohort were visited once. For each study visit, a questionnaire was administered and a blood sample was collected by finger prick for a malaria rapid diagnostic test (RDT) [38].

### 2.2. GPS data loggers

Criteria for selection of the GPS devices were developed to accommodate the study population, ensure participants would not be responsible for charging the devices and to protect privacy. These criteria included size, weight, water resistance, battery life, memory size, programming capabilities, motion detection and validity. IgotU® GT-600 (Mobile Action Technology) GPS loggers were selected as they were shown to be accurate (point accuracy of 4.4 m and line accuracy of 10.3 m) and acceptable in a study conducted in Iquitos, Peru [22]. These devices were light weight (37 g), had large battery capacity (750 mAh), were programmable, could collect up to 262 000 waypoints with 64 Mb of memory and were water resistant [22–24]. The loggers could be password-protected and accessed only with the accompanying software when connected to a computer with a custom USB cable. The data loggers could be worn using a Velcro strap or lanyard, or carried in a pocket or a bag, with the only requirement that they be carried with the participant continuously during their normal daily movement. As the devices were motion activated, they could be removed when participants were sleeping or sedentary to preserve battery life.

All participants enrolled in the existing longitudinal cohort who were 13 years and older were invited to participate during bi-monthly study visits. A non-random convenience sample was selected during study visits from October 2013 through to August 2014. The study staff aimed to enrol 12 participants per month and have at least 10 complete the full month. Up to three participants per household were permitted to participate with no more than two individuals participating concurrently in a single month. Enrolled participants were requested to carry the GPS data logger at all times they were active for a one-month period. This allowed for a full year of data collection to assess seasonal movement patterns.

Serial numbers of the GPS data loggers were matched to participant unique identification numbers. The power button was locked and the GPS data loggers were password-protected, so that only study staff could access the data. Geographical position was logged every 2.5 min. The loggers were programmed to be motion activated and hibernate when not in motion to conserve battery life. To prevent data loss owing to limited battery life, participants carried one logger for two weeks at which time the device was exchanged for a fully charged logger during a household visit by the study team.

Data collected from the loggers contained date, time, longitude and latitude. Study staff maintained a monthly record of the date and time the devices were distributed and collected.

The observed rainfall collected at the study site using a HOBO weather station (Onset Computer Corporation, Bourne, MA, USA) was graphed to document seasonal rainfall patterns.

### 2.3. Data management

After each two-week collection period, data were downloaded from each device using the @trip software (Mobile Action Technology, Inc., New Taipei City, Taiwan). The unique participant and household identification numbers were added manually. The data were checked for inconsistent logging and device errors, such as battery failure or unrealistic locations. Raw data for each two-week period were uploaded to a secure REDCap (Research Electronic Data Capture) server [39]. The monthly record was used to remove data points where the logger was in transit with study staff to and from study households.

### 2.4. Mapping movement patterns

Movement data coordinates were projected into UTM Zone 35S, WGS 1984 and imported into ArcGIS (ESRI 2012. ArcGIS Desktop: Release 10.2. Environmental Systems Research Institute, Redlands, CA, USA) for pre-processing and analysis. Pre-processing was done by removing erroneous data points based on unrealistic changes in shape, speed or direction in the movement tract using a software extension developed for GPS-based trajectory analysis in ArcScene by Qi & Du [28]. The cleaned and pre-processed movement tracts were used to determine the cumulative amount of time spent at each location. High-resolution movement density maps were created by estimating the spatial density of travel paths. The spatial densities were estimated non-parametrically using the kernel density approach in the ArcScene software extension [28] with a bandwidth fixed at 100 m. This bandwidth was chosen based on the reported error and spatial resolution of the GPS loggers and to optimize visualization of the maps created. The resulting movement density maps characterize high and low areas of movement and are used as an estimate of the population space used per month.

The movement density maps created to display the movement trajectory density for each participant were overlaid on a satellite image of the study area with the enumerated households to represent the cumulative amount of time each participant spent in different areas. Density maps for participants were aggregated up to the month of collection to visually evaluate seasonal trends in movement patterns. For each month, movement densities were normalized based on the overall range to have all months on the same scale for comparisons. Short- and long-distance movement patterns were evaluated by overlaying the respective density maps on the satellite imagery. Long-distance movements were defined as those that left the study area and short-distance movements those that did not leave the study area. A three-dimensional density map, with the third dimension representing the amount of time spent at a location, was created and overlaid on a previously published malaria risk map of the study area [38] to visualize movement patterns in and out of areas of higher and lower malaria risk.

### 2.5. Calculating activity space

To create movement trajectories for each participant, time was converted from date, hour, minute and second format to a numeric format. The total time participants carried a GPS data logger for each two-week time period was calculated independently to permit inclusion of individuals who only carried the logger for the first two-week period or experienced battery failure during a two-week period. The time elapsed between two consecutively logged geographical locations was then calculated. To account for differences in the total amount of time recorded by the GPS data logger for each participant, the proportion of time spent in each logged geographical location was calculated. These movement trajectories, containing logged geographical locations with calculated proportions of time spent in each location, were spatially joined to the locations of participants’ households. The proportion of time and density of time spent in logged geographical locations were plotted against distance from the household to determine the distribution of movement patterns and activity space in relation to the household compound.

The proportion of time participants spent within their household compound was also calculated. A typical household compound in the study area has one or more domestic structures with several smaller structures, such as cooking houses or animal kraals. A household compound was defined as a grouping of these structures that function as a family unit. During each study visit, geographical coordinates of the household were collected from the front entrance of the main domestic structure using a tablet computer. To account for the household compound layout, and error owing to the limits of spatial resolution of the GPS logger and the tablet used to collect the GPS coordinates, the household was defined as a 100 m circular buffer around the measured household coordinates. Participant movement trajectories were spatially joined to the household buffer. This allowed for the movement to be defined as being at or away from the household compound. The proportion of time (median and interquartile range (IQR)) spent away from the household was plotted by month to determine if there was a seasonal trend. A Kruskal–Wallis test was used to determine if there were statistically significant differences between the median proportions of time spent away from the household by month.

As the primary vector, *A. arabiensis*, is known to have exophilic feeding behaviour, the amount of time spent away from the household compound during peak biting times was estimated [32,33]. We were not able to estimate the time outdoors owing to the complex household structure and spatial resolution of the GPS loggers. Peak biting times for *A. arabiensis* were estimated to be between 19.00 and 6.00 hours in the study area [33]. First, the proportion of time each participant was at and away from the household compound was plotted by month to determine seasonal patterns in time spent away from the household compound. The movement trajectories for each participant were then stratified by within and outside peak vector biting times. The subset of trajectories during peak biting times was used to calculate the proportion of time spent away from the household compound during peak biting times and graphed by month to determine seasonal patterns. The Kruskal–Wallis test was used to determine if there were statistically significant differences between the median proportions of time spent away from the household during biting times by month.

The proportion of time spent in areas of high malaria risk was calculated using a previously constructed malaria risk map [38]. The risk map was created using community-based surveys and environmental features obtained from satellite imagery and remotely sensed data to predict the probability of malaria infection in the study area over 21 months in 2007 and 2008 [38]. The resultant risk map estimated the ecological risk of malaria infection (including subclinical infection) confirmed by RDT as the predicted probability of infection [38]. Areas were categorized as being high (probability of infection 0.50 and greater) and low (probability of infection less than 0.50) malaria risk based on this map. Polygons of areas of high and low malaria risk were created from the raster formatted malaria risk map. The participants’ movement trajectories were spatially joined to the high malaria risk polygons. Participants were stratified by household compound location as being within an area of high or low malaria risk. The proportion of time spent in areas of high malaria risk was calculated stratified by participants residing in high and low malaria risk areas, and aggregated by month to assess seasonal patterns. The proportion of time spent in high-risk areas at and away from the household compound was calculated for participants residing in areas of high malaria risk. Only the proportion of time spent away from the household compound was calculated for participants residing in areas of low malaria risk, as they did not have the opportunity to spend time in an area of high malaria risk within their household compound. Statistically significant differences in proportions of time spent in high malaria risk areas comparing participants residing in high- and low-risk areas were tested using the Kruskal–Wallis test.

## 3. Results

Two hundred and twenty individuals from 49 households were included in the full longitudinal cohort. During the study period, 173 eligible participants from 30 households in this longitudinal cohort were invited to participate in the study, of whom 69 agreed to carry a GPS data logger. All participants completed the first two weeks of data collection and 62 completed the second two weeks. Data from one participant were excluded from analysis as they reported they were ill and gave the GPS data logger to other family members. The other six participants who did not complete the second two weeks declined further participation. The GPS data loggers were well accepted among participants and even became popular within the community. The age distribution of participants varied slightly by month (range 19–55 years; *p* = 0.04), but there were no differences by sex (*p* = 0.71) (table 1). The convenience sample of participants was older than the eligible study population (median age 39 years versus 24 years; *p* = 0.01) but not different by sex (*p* = 0.85) (table 1).

**Table 1.** Demographic characteristics of the study participants by month and comparison with the remaining eligible population.

	number	age in years (median (IQR))	per cent male (% (95% CI))
study participants	68	39.1 (19.8–54.7)	50.0 (38.0–62.0)
Oct	12	54.5 (42.6–61.7)	41.7 (12.0–71.3)
Dec	12	45.8 (20.3–57.7)	50.0 (20.0–80.0)
Feb	11	19.3 (16.6–51.4)	36.4 (6.0–66.7)
Apr	11	39.0 (23.2–41.6)	54.6 (23.1–86.0)
June	12	40.8 (26.9–48.2)	50.0 (20.0–80.0)
Aug	10	21.5 (14.5–30.2)	70.0 (39.5–100.0)
remaining eligible individuals	105	23.8 (16.0–42.8)	48.6 (38.9–58.2)

**Figure 1.**
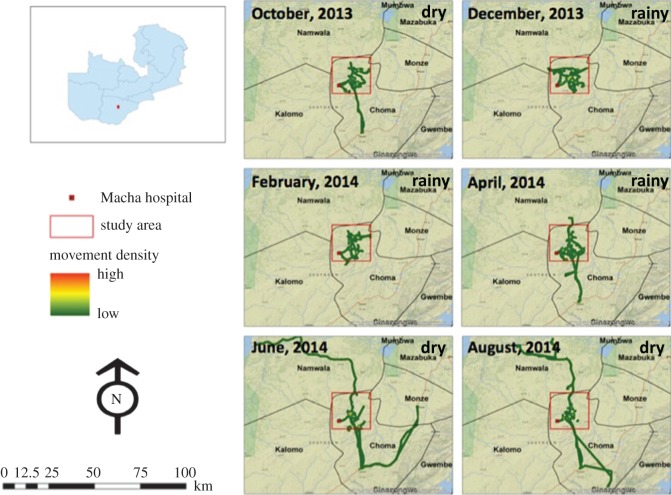
High-resolution maps showing long-distance movement density in the study area in southern Zambia from October 2013 to August 2014 overlaid on a National Geographic, ESRI Worldwide base map. Long-distance movements are those that extend beyond the 1200 km^2^ study area outlined in red. The rainy season includes December, February and April, with the dry season during October, June and August.

The long-distance movement density maps, which display movement patterns outside the study area, showed visual evidence of seasonal patterns in population movement (figure 1). There was less long-distance movement during the rainy season (December and February), with no participants leaving the study area (figure 1). From April through to August, long-distance movement increased as participants travelled outside the study area and stayed further from home for longer periods (figure 1).

**Figure 2.**
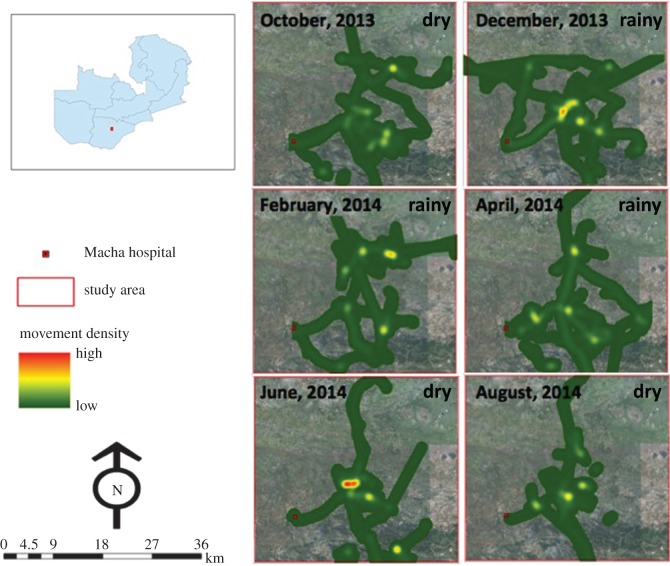
High-resolution maps showing short-distance movement density in the study area in southern Zambia from October 2013 to August 2014 overlaid on a Quickbird high-resolution satellite image of the study area. Short-distance movements are those within the 1200 km^2^ study area outlined in red. The rainy season includes December, February and April, with the dry season during October, June and August.

**Figure 3.**
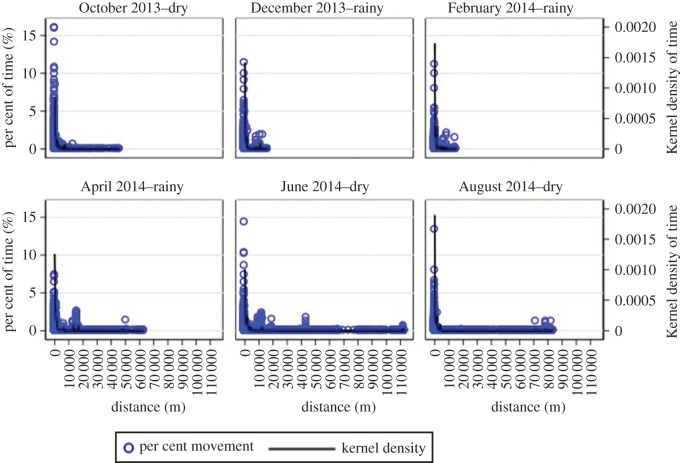
Per cent of time spent in locations by distance from the participant's home for each month from October 2013 to August 2014. The rainy season includes December, February and April, with the dry season during October, June and August.

The short-distance maps, which display high-resolution movement patterns within the 1200 km^2^ study area, did not show visual evidence of seasonal mobility patterns (figure 2). Visual evidence of seasonal patterns in long-distance but not short-distance movement from the density maps was supported by density plots of the proportion of movement trajectory by distance from the household compound (figure 3) and showed longer trips farther from home beginning in April as the rainy season ended (figure 3). These density plots confirmed that participants spent most of their time close to their household compound with seasonal, longer trips that included shorter movements around these distant locations (figure 3). During December and February, only one trip farther than 20 km from the participant's home was recorded. However, from April through to August, a total of 14 trips farther than 20 km from participants’ homes were recorded (four in April, seven in June and three in August). This seasonal pattern of increased long-distance movement following the end of the rainy season coincided with the typical seasonal peak in clinical malaria cases, and the measured monthly rainfall was consistent with the expected seasonal rainfall patterns.

**Figure 4.**
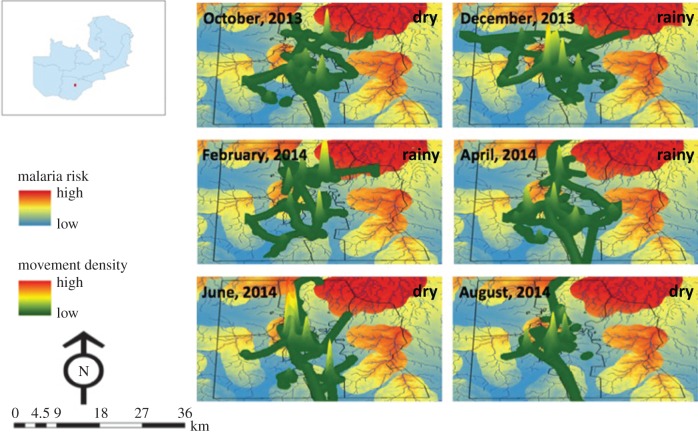
Three-dimensional maps showing short-distance movement densities within the 1200 km^2^ study area overlaid on a malaria risk map [38] to display movement in areas of varying malaria risk for each month from October 2013 to August 2014. Lowest risk is indicated by blue, intermediate risk in yellow and highest risk in red. The rainy season includes December, February and April, with the dry season during October, June and August.

**Figure 5.**
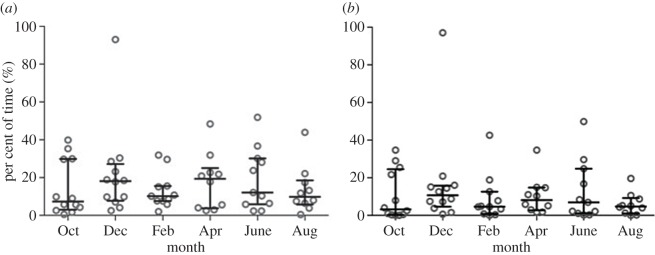
Per cent of time spent away from the household compound by month from October 2014 to August 2014. (*a*) All time periods; (*b*) restricted to *A. arabiensis* biting hours (19.00–06.00).

Movement density in areas of high and low malaria risk was mapped (figure 4). These maps indicated that participants spent most of their time at a limited number of local sites (figure 4). There was no evidence of a seasonal trend in the percentage of time spent away from the household compound (figure 5*a*), with a median of 10.6% (IQR: 5.8–23.8) of time spent away from the household compound. This decreased by nearly half during peak anopheline biting times to a median of 5.6% (IQR: 1.7–14.9) of time spent away from the household compound (figure 5*b*).

**Figure 6.**
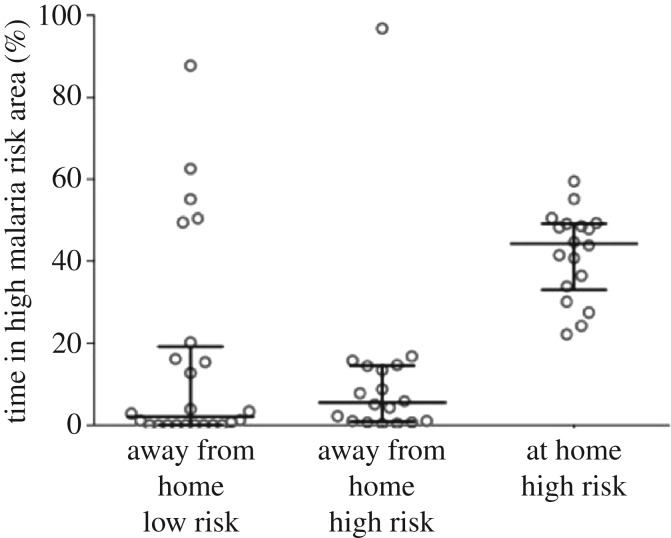
Per cent of time spent away from the household compound for all months (October 2013–August 2014) combined during *A. arabiensis* biting times (19.00–06.00) in areas of high malaria risk stratified by household location (high or low malaria risk area).

The amount of time spent in areas of high malaria risk was dependent on whether the household compound was in an area of high malaria risk (figure 6). The per cent of time spent in areas of high malaria risk for participants residing in areas of high malaria risk ranged from 83.2% to 100% (median: 96.4%, IQR: 91.1–98.1), and the per cent of time spent in areas of high malaria risk for participants residing in areas of low malaria risk ranged from 0% to 36.7% (median 0%, IQR: 0.0–0.66).

The amount of time spent in high-risk areas away from the household compound during peak vector biting times was not different between participants residing in household compounds in high malaria risk (median: 5.5%, IQR: 0.98–14.4) and low malaria risk (median: 2%, IQR: 0–18.2) (*p* = 0.4) areas (figure 6). During peak biting times, the median time spent within the household compound in high malaria risk areas was 44.3% (IQR: 33.9–49.2), higher than the amount of time spent away from the household compound during peak biting times in both high-risk areas (5.4% (IQR: 0.98–14.4)) and low-risk areas (2.0% (IQR: 0.0–18.2)) (*p* = 0.001) (figure 6).

## 4. Discussion

Residents of rural, southern Zambia primarily spent time close to their household compound, with frequent short movements around their household and infrequent longer trips to distant locations that included shorter movements around these locations. Long-distance movement patterns showed clear seasonality. During the rainy season, participants did not travel far from their household compound, presumably to stay closer to their farms but perhaps also because roads became impassable. As the rainy season ended, participants began to travel further from their household compound and stayed there for longer durations.

The convenience sample of 69 participants for this study was small; however, a large amount of data was generated on movement patterns over a 1 year period that included both rainy and dry seasons. The demographic characteristics of this sample was older but resembled eligible participants in the parent longitudinal cohort. As the longitudinal cohort is a representative sample, the movement patterns observed in the GPS logger study are probably representative of adults in the underlying population. Additionally, these patterns are probably generalizable to similar rural, agrarian populations in sub-Saharan Africa.

The long-distance movement patterns at the end of the rainy season and during the dry season seemed to be consistent with long-distance foraging patterns used when searching for heterogeneously distributed food [31,40]. This pattern consists mainly of shorter movements (e.g. frequent short-term trips close to home) combined with fewer farther movements (e.g. infrequent longer-term trips far from home) [31,41]. Importantly, participants in this study returned to the same home, which is not typical of many long-distance foraging patterns. While movement patterns are important for understanding malaria epidemiology, challenges remain as to how best to incorporate these patterns into malaria transmission models [42]. Specifically, limited movement away from the home during the rainy season, increased long-distance travel after the rainy season and minimal mixing between ecologically high and low malaria risk areas are important factors in modelling micro-scale malaria transmission and planning elimination strategies.

Malaria prevalence has declined dramatically in parts of southern Zambia, but the region remains receptive to malaria transmission and clinical cases typically occur each year throughout the rainy season, increasing at the end of the rainy season in April. This seasonal increase in clinical malaria cases coincides with increased population mobility. While malaria prevalence is low in the study area, some surrounding areas have higher malaria prevalence. Movement to these areas for extended periods and travel back home may result in imported infections. However, this may only be important at the end of the rainy season in the month of April, as this marks the beginning of the dry season when vector populations are insufficient to maintain transmission. Thus, these long-distance, seasonal movement patterns may result in imported infections at the end of the rainy season but are unlikely to facilitate transmission during the dry season.

Participants spent approximately 5% of time away from their household compound during peak biting times. However, the spatial resolution of the GPS data loggers and satellite imagery limited the ability to determine if participants were inside a domestic structure. The malaria risk map used to determine areas of high and low risk was developed when malaria prevalence was approximately 9%, and parasite prevalence subsequently dropped to less than 1% at the time of this study. However, as the map predicted malaria risk based on ecological features, qualitatively these findings remain valid for determining areas of high and low malaria risk. For short-distance movement patterns, the proportion of time spent in areas of high malaria risk was strongly dependent on whether the participant's household compound was in an area of high malaria risk. Individuals who resided in areas of higher and lower malaria risk did not spend much time in areas of the opposite risk. However, even a small amount of time spent in a high malaria risk area could result in infection and the introduction of parasites into low malaria risk areas, propagating local transmission. Therefore, malaria elimination interventions implemented at the household level, such as insecticide-treated nets, indoor residual spraying and reactive case detection, may benefit from the less frequent, long-distance movement during the rainy season.

## 5. Conclusion

The human mobility patterns observed in this study can be described as circulatory rural–rural movement [1], although these GPS quantitative estimates of movement provide more information about the frequency of travel and measures of risk. These movement patterns suggest how malaria elimination efforts could be threatened through movement of uninfected residents to higher malaria risk areas and transmitting parasites to vectors upon returning home [1,8]. These types of movement patterns and their seasonality should be considered when planning malaria elimination strategies. Because of restricted mobility during the rainy season, interventions directed at households may be more effective. In areas at higher ecological risk, interventions could be targeted at households during the rainy season, as mobility outside of high-risk areas during this time is minimal.

## Permission to carry out fieldwork

Permission to carry out fieldwork in Zambia was obtained and approved by the Institutional Review Board at the Johns Hopkins Bloomberg School of Public Health, and the Review Board and the Tropical Diseases Research Center Ethics Review Committee in Ndola, Zambia.

## Data accessibility

Data used in this study have not been made publically available. It is the policy of the Ministry of Health of Zambia to not publically release health data collected within the country. Furthermore, owing to the nature of these high-resolution movement data, making them publically available may potentially breach the confidentiality and anonymity of participants. Individuals may request access to the data for the purposes of reproducibility and additional investigatory analyses by contacting the corresponding author at ksearle1@jhu.edu.
